# Glucose-limiting conditions induce an invasive population of MDA-MB-231 breast cancer cells with increased connexin 43 expression and membrane localization

**DOI:** 10.1007/s12079-020-00601-3

**Published:** 2021-02-16

**Authors:** Jennifer C. Jones, Amanda M. Miceli, Mary M. Chaudhry, Chloe S. Kaunitz, Mallika A. Jai, Romel N. Pancho, Alan Lazzar, Bradley S. Taylor, Vishnupriya Bodempudi, Prarthana P. Jain, Sheeri Hanjra, Alexander E. Urban, Brian Zanotti, Ellen K. Kohlmeir, Thomas M. Bodenstine

**Affiliations:** 1 Department of Biochemistry and Molecular Genetics, Midwestern University, 555 31st Street, Downers Grove, IL 60515 USA; 2 Chicago College of Pharmacy, Midwestern University, 555 31st Street, Downers Grove, IL 60515 USA; 3 Biomedical Sciences Program, Midwestern University, 555 31st Street, Downers Grove, IL 60515 USA; 4 Chicago College of Osteopathic Medicine, Midwestern University, 555 31st Street, Downers Grove, IL 60515 USA; 5 Department of Microbiology and Immunology, Midwestern University, 555 31st Street, Downers Grove, IL 60515 USA; 6 Core Facilities, Midwestern University, 555 31st Street, Downers Grove, IL 60515 USA; 7 College of Health Sciences, Midwestern University, 555 31st Street, Downers Grove, IL 60515 USA; 8 College of Graduate Studies, Midwestern University, 555 31st Street, Downers Grove, IL 60515 USA

**Keywords:** Connexin, Gap junction, Breast cancer

## Abstract

Gap junctional intercellular communication (GJIC) is a homeostatic process mediated by membrane channels composed of a protein family known as connexins. Alterations to channel activity can modulate suppression or facilitation of cancer progression. These varying roles are influenced by the cancer cell genetic profile and the context-dependent mechanisms of a dynamic extracellular environment that encompasses fluctuations to nutrient availability. To better explore the effects of altered cellular metabolism on GJIC in breast cancer, we generated a derivative of the triple-negative breast cancer cell line MDA-MB-231 optimized for growth in low-glucose. Reduced availability of glucose is commonly encountered during tumor development and leads to metabolic reprogramming in cancer cells. MDA-MB-231 low-glucose adapted cells exhibited a larger size with improved cell–cell contact and upregulation of cadherin-11. Additionally, increased protein levels of connexin 43 and greater plasma membrane localization were observed with a corresponding improvement in GJIC activity compared to the parental cell line. Since GJIC has been shown to affect cellular invasion in multiple cancer cell types, we evaluated the invasive qualities of these cells using multiple three-dimensional Matrigel growth models. Results of these experiments demonstrated a significantly more invasive phenotype. Moreover, a decrease in invasion was noted when GJIC was inhibited. Our results indicate a potential response of triple-negative breast cancer cells to reduced glucose availability that results in changes to GJIC and invasiveness. Delineation of this relationship may help elucidate mechanisms by which altered cancer cell metabolism affects GJIC and how cancer cells respond to nutrient availability in this regard.

**Supplementary material:**

The online version of this article (10.1007/s12079-020-00601-3) contains supplementary material, which is available to authorized users.

## Introduction

The development and progression of cancer is affected by genetic alterations within cancer cells and an ever-changing microenvironment that influences their behavior. Limitations in nutrient availability are common during tumor growth in hypoxic regions of primary tumors and colonization at metastatic sites as cancer cells encounter new tissue microenvironments (Simoes et al. 2015; DeBerardinis and Chandel 2016). In particular, heterogenous blood perfusion within tumors leads to decreased oxygen and glucose levels and a reprogramming of cellular signaling which creates new cellular phenotypes optimized for growth in these conditions (Vaupel et al. 1989; Gillies et al. 1999; Garcia-Jimenez and Goding 2019). The ability of cancer cells to adapt to these changing conditions increases their potential to survive and proliferate. This often requires utilization of secondary fuel sources for nitrogen and carbon and necessitates modifications to biochemical pathways (DeBerardinis and Chandel 2016; Pavlova and Thompson 2016).

Gap junctions are intercellular channels mediated by a family of proteins known as connexins and support cellular homeostasis by allowing the regulated passage of ions, nutrients and signaling molecules (Nielsen et al. 2012). This gap junctional intercellular communication (GJIC) is often dysregulated in cancer where it is frequently decreased due to downregulation of connexin genes, mislocalization of connexin proteins and changes to cellular adhesion junctions (Aasen et al. 2016). Numerous reports have demonstrated a causative relationship between the loss of GJIC and acquisition of cancer cell hallmarks or suppression of these features following restoration of GJIC (Mehta et al. 1991; King and Lampe 2004; Shao et al. 2005; Wang et al. 2014). However, other data have established that GJIC can also promote aggressive qualities in cancer cells (Bates et al. 2007; Ghosh et al. 2014; Hong et al. 2015; Zibara et al. 2015). Multiple models have demonstrated connexin expression and/or GJIC to be correlated with metastasis (Lamiche et al. 2012; Ogawa et al. 2012; Tang et al. 2013). Moreover, cancer cell gap junctions form between endothelial cells during metastasis, as well as stromal cells at secondary metastatic sites (el-Sabban and Pauli 1994; Chen et al. 2016). Thus, GJIC remains a dynamic process in the cancer cell, consequences of which are influenced by context-dependent mechanisms that may suppress or promote cancer cell survival and function.

An increased understanding of the role GJIC plays in cancer cell metabolism is providing important information for how gap junctions mediate the transfer of metabolites in the tumor microenvironment (Contreras et al. 2002; Dovmark et al. 2017, 2018). Conversely, to better understand the role of metabolism in regulation of GJIC, we evaluated changes in the triple-negative breast cancer cell line MDA-MB-231 when these cells adapted to low-glucose availability, a variant we refer to as MDA-MB-231^LG^. This derivative exhibited increased connexin protein expression and GJIC. Additionally, these cells exhibited highly invasive qualities that were affected, in part, by inhibition of GJIC.

## Materials and methods

### Antibodies

Antibodies targeting connexin 43, actin, vimentin, N-cadherin, E-cadherin, claudin-1, cadherin-11 (immunofluorescence), β-catenin, LC3 and horse radish peroxidase conjugated secondary antibodies were purchased from Cell Signaling (Danvers, MA, USA). Cadherin-11 antibody (western blot) and Alexa fluor conjugated secondary antibodies for immunofluorescence were purchased from Thermo Fisher Scientific (Waltham, MA, USA). GAPDH antibody was purchased from EMD Millipore. HIF1α antibody was purchased from BD Biosciences.

### Cell lines and culture

MDA-MB-231 (HTB-26), Hs578T (HTB-126), MCF-7 (HTB-22) and T47D (HTB-133) cell lines were purchased from the American Type Culture Collection (ATCC; Manassas, VA, USA) and maintained in RPMI 1640 supplemented with 10% fetal bovine serum (FBS). Cells were grown in a humidified incubator at 37 °C, 5% CO_2_ and passaged using trypsin–EDTA. Routine screening for mycoplasma was performed via PCR detection using the Promokine PCR Mycoplasma Test Kit I/C (PromoCell, Heidelberg, Germany). MDA-MB-231^LG^ were grown in RPMI 1640 glucose-free media supplemented with 10% FBS. All experiments involving parental and LG cells were performed in the respective media unless otherwise indicated. All cells were validated by short tandem repeat (STR)-analysis at the Northwestern University NUSeq Core Facility (Chicago, IL, USA) and compared to ATCC cell identification data for verification (Table 1, Supp. Table 1).

**Table 1:** STR analysis of MDA-MB-231 and MDA-MB-231^LG^

Marker	MDA-MB-231	MDA-MDB-231^LG^
Amelogenin*	X	X
D3S1358	16	16
D1S1656	15, 17	15, 17
D2S441	14, 15	14, 15
D10S1248	14, 16	14, 16
D13S317*	13	13
Penta E	11	11
D16S539*	12	12
D18S51	11, 16	11
D2S1338	20, 21	20, 21
CSF1PO*	12, 13	12, 13
Penta D	11, 14	11, 14
TH01*	7, 9.3	7, 9.3
vWA*	15, 18	15, 18
D21S11	30, 33.2	30, 33.2
D7S820*	8, 9	8, 9
D5S818*	12	12
TPOX*	8, 9	8, 9
D8S1179	13	13
D12S391	17, 18	17, 18
D19S433	11, 14	11, 14
FGA	22, 23	22, 23
D22S1045	16	16

Asterisk indicate 9 markers defined by ATCC criteria for 100% match

### Proliferation and viability

Cellular proliferation and viability were measured by use of CellTiter 96 AQueous One Solution reagent (EMD Millipore) on a PerkinElmer Enspire Multimode plate reader (Waltham, MA, USA) according to manufacturer’s protocols. CellEvent Green Caspase 3/7 and SYTOX-AADvanced reagents (Invitrogen, Carlsbad, CA, USA) were used to assess caspase activity and cell integrity using a Beckman Coulter (Brea, CA, USA) CytoFLEX flow cytometer with CytExpert analysis software. Positive controls for CellEvent reagent were demonstrated with staurosporine (Caspase 3/7) and heat killing (SYTOX) (Supp. Fig. 1).

### pH readings

Cells were plated at equal densities and allowed to grow for 96 h without media changes. At 96 h media from wells was removed and filtered through a 0.22 µm sterile filter and read on a Mettler-Toledo SevenEasy pH meter.

### Scanning electron microscopy

Cells were grown on glass coverslips and fixed with 4% paraformaldehyde at 4 °C for 24 h. Cells were then washed with phosphate-buffered saline (PBS), dehydrated with increasing concentrations of ethanol (30%, 50%, 70%, 80%, 95% and 100% for 10 min each at 4 °C), and dried in a Leica (Wetzlar, Germany) EM CPD300 critical point dryer. Samples were then sputter-coated with 10 nm silver using a Leica EM ACE600. Cells were visualized with a JEOL (Akishima, Tokyo, Japan) JCM-6000Plus scanning electron microscope at 5 kV and 10 kV.

### Fluorescence microscopy and GJIC analysis

For immunofluorescence, cells were grown on glass coverslips in 24-well plates, washed with ice cold PBS and fixed in ice cold 100% methanol at − 20 °C for 20 min. Cells were blocked in 2% bovine serum albumin (BSA) in PBS and incubated with indicated antibodies. For GJIC analysis, cells were analyzed by the method of Goldberg and quantified by flow cytometry (Goldberg et al. 1995; Czyz et al. 2000). Briefly, donor cells were labeled with 5 µM of the lipophilic permanent dye CM-DiI (Invitrogen) and 10 µM calcein-AM (Invitrogen) and cultured with non-labeled cells. Spread of calcein from donor cells (CM-DiI/calcein positive) to acceptor cells (calcein only) indicated GJIC activity as demonstrated with the highly gap junction coupled Hs578T breast cancer cell line (Supp. Fig. 2a). Images for immunofluorescence and GJIC analysis were obtained with a Leica DMi8 fluorescent microscope equipped with a Leica DFC 9000GT camera and X-Cite XLED1 fluorescence source (Lumen Dynamics, Mississauga, Ontario, Canada). Quantification of GJIC was performed using a BD Biosciences (San Jose, CA, USA) FACSCalibur flow cytometer with CellQuest Pro software using the assay described above with 0.5 µM Cell Tracker Deep Red (Invitrogen) and 0.75 µM calcein-AM (Supp. Fig. 2b). Coupling efficiency was represented as the level of GJIC activity and calculated as acceptor cells/donor cells and normalized to control.

### Matrigel invasion assays

Growth factor reduced (GFR) Matrigel basement membrane matrix (Corning, NY, USA) was utilized for two different three-dimensional cell culture assays. For the embedded assay, Matrigel was diluted from 8 to 5 mg/ml using RPMI 1640 with or without glucose with 10% FBS before 300 μl was added to each well. 30 μl of 1 × 10^6^ cells/ml were added to 270 μl of diluted Matrigel and added on top of the first layer. After approximately 30–45 min of incubation, 500 μl of appropriate medium was added to the Matrigel layers. For the on-top assay, 300 μl of Matrigel was added directly to the wells without dilution. The Matrigel layer was allowed to solidify at 37 °C and 5% CO_2_ for 45 min and 250 μl of 25,000 cells/ml were plated directly on top of the first Matrigel layer and allowed to incubate for 45 min. 25 μl of Matrigel was mixed with 225 μl of the appropriate medium and added to the wells. Cells were then incubated for 6 days for each assay with medium changed at 48 h.

Growth factor reduced Matrigel invasion chambers (Corning) were used for the Matrigel invasion assay. 500 μl of warm serum-free medium (± 2 mM glucose) was added to each transwell insert for 2 h in the incubator to rehydrate the Matrigel. Cell suspensions were made at a concentration of 100,000 cells/well in serum-free medium. Medium in inserts was removed after 2 h before cell suspensions were added to each insert. 750 μl of 5% FBS medium was added as a chemoattractant to the bottom of wells in the 24-well plate. The invasion assay was incubated for a total of 24 h. A sterile cotton swab was used to remove non-invasive cells from the membrane. Pre-chilled 100% methanol was used as a fixative at 500 μl per insert for approximately 20 min on an orbital shaker at 4 °C. Invading cells were stained with crystal violet or mounted to glass slides using Fluoroshield with DAPI (Thermo Fisher Scientific) for quantification and imaged using a Nikon (Minato, Tokyo, Japan) Eclipse Ti2 microscope with Fi3 camera. Quantification of DAPI was performed using NIS Elements AR software.

### Western blot analysis

Whole cell lysates were collected using lysis buffer containing 25 mM Tris, pH 7.4, 5% glycerol, 1% SDS and 1× protease and phosphatase inhibitors (Thermo Fisher Scientific). Lysates were passaged through a 21-gauge needle 10× on ice, combined with Laemmli sample buffer supplemented with β-mercaptoethanol and heated at 95 °C for 10 min. Samples were separated using 12% SDS-PAGE, transferred to PVDF and detected by enhanced chemiluminescence using a Bio-Rad Gel Imager (Hercules, CA, USA). Blocking and antibody solution consisted of 5% non-fat dry milk in tris-buffered saline with 0.1% tween-20. HEK293T connexin 43 overexpression lysate and empty vector control were purchased from Novus Biologicals. Additional cell lines were used as positive controls for some proteins and analyzed under the same conditions listed above. Hs578T, N-cadherin (Hazan et al. 1997; Nieman et al. 1999); MCF7, E-cadherin (Nieman et al. 1999; Pishvaian et al. 1999); T47D, claudin-1 (Majer et al. 2016; Mattern et al. 2019). Densitometry was performed using NIH ImageJ software and proteins of interest normalized to actin.

### Statistical analysis

Analysis was performed using GraphPad Prism 8. Data are presented as mean ± standard deviation (SD) and significance determined using unpaired student *t* test analysis. Differences were considered statistically significant at *p* < 0.05 (*).

## Results

### Characterization of MDA-MB-231^LG^

**Fig. 1:**
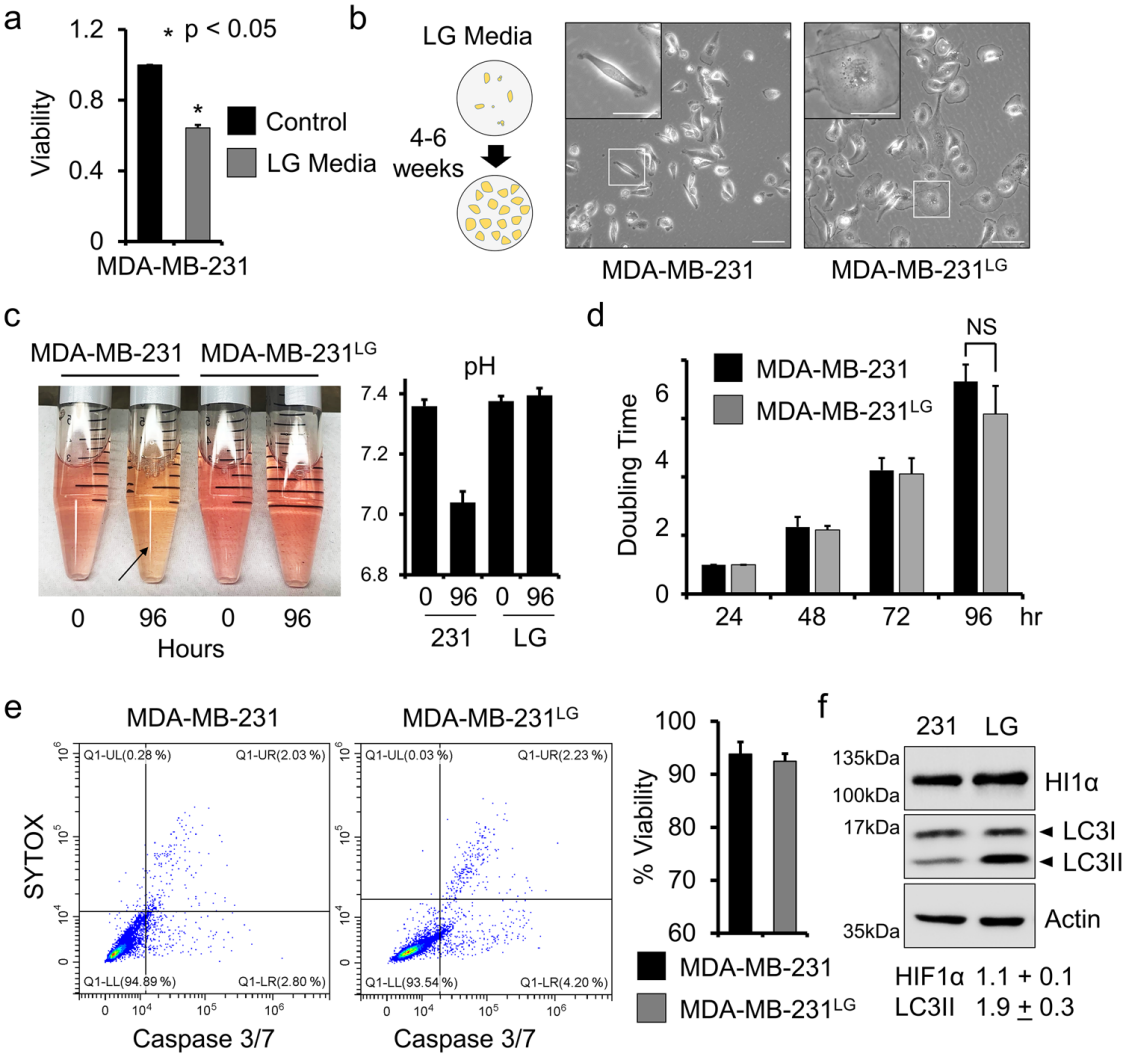
Characterization of MDA-MB-231^LG^. **a** MTT assay demonstrating viability of MDA-MB-231 cells grown in control or low-glucose (LG) media for 72 h. Experiments were performed in triplicate three independent times. Results were normalized to control and represented as fold change. Data represent the mean ± SD. (*p < 0.05) **b** Phase microscopy of MDA-MB-231 and MDA-MB-231^LG^ demonstrating differences in morphology and size. Scale bar: 100 µm; inset: 50 µm. **c** Cell lines were grown in respective media (control, LG) for 96 h. Time 0 represents unused media at start of experiment. Arrow indicates acidification based on color of phenol-red in culture media. Quantification readings were performed in triplicate and data represent the mean of three independent experiments ± SD. **d** Doubling-times for each cell line at 48, 72 and 96 h. Experiments were performed in triplicate and represent the mean of three independent experiments ± SD. (NS, not significant) **e** Evaluation of cellular viability by flow cytometry using markers for cleavage of caspase-3 (x-axis) to indicate apoptosis and SYTOX (y-axis) as a marker of cellular integrity/necrosis. Data was quantified and presented as % viability. Experiments were performed in triplicate and data represent the mean of two independent experiments ± SD. **f** Representative western blot analysis of three independent experiments for HIF1α and LC3. Densitometry represents fold-change ± SD in MDA-MB-231^LG^ compared to MDA-MB-231. β-actin used as a loading control. **b–f** MDA-MB-231 cultured in control media; MDA-MB-231^LG^ cultured in low-glucose media

To examine the effects of metabolic changes on GJIC in breast cancer cells, we grew the MDA-MB-231 cell line in RPMI 1640 without glucose and supplemented with 10% FBS. Because trace amounts of glucose are present within FBS, we did not consider this media glucose-free. However, based on manufacturer’s specifications, final glucose concentration in the prepared media were below 0.130 mM. This accounted for a greater than 15× fold reduction in available glucose compared to control RPMI 1640 containing 2 mM glucose. Growth in this media decreased viability within 2–4 days of culture when evaluated using an MTT assay (Fig. 1a). To generate a population of cells optimized for growth in these conditions, we plated MDA-MB-231 at low density (20–30% confluence) and maintained surviving cells for 4–6 weeks. Media was replaced every 2 days to remove apoptotic cells and cellular debris. Small populations of surviving cells initially exhibited quiescence followed by restoration of cellular proliferation. To avoid clonal selection, surviving populations from multiple plates were combined and propagated. The growth pattern of these cells normalized and regular maintenance was initiated using continued culture in low-glucose media. The surviving population exhibited morphology that was noticeably different from the parental MDA-MB-231 (Fig. 1b). Cells were larger with rounded membranes, distinctly different from the mesenchymal morphology of the parental MDA-MB-231. These cells were designated MDA-MB-231^LG^ for their ability to grow in low glucose conditions. MDA-MB-231^LG^ did not acidify low-glucose culture media compared to parental cells grown under standard conditions as evidenced by the effects on phenol-red (Fig. 1c). Analysis of cellular proliferation showed a similar growth pattern (Fig. 1d). Cells appeared healthy with minimal signs of cellular stress. To confirm this, we assessed markers for apoptosis (caspase-3/7 cleavage) and overall cellular integrity (SYTOX) by flow cytometry demonstrating greater than 90% viability in low-glucose media similar to that of MDA-MB-231 grown in control media (Fig. 1e). As our growth conditions involved the reduction of glucose but did not affect oxygenation, we evaluated levels of LC3II as a marker of autophagy and hypoxia inducible factor α (HIF1α) as an indicator of response to hypoxic conditions. Indeed, greater levels of LC3II were present in the MDA-MB-231^LG^ while HIF1α remained relatively unchanged (Fig. 1f).

Since the MDA-MB-231^LG^ displayed considerably different morphology, we verified that the MDA-MB-231^LG^ were a true derivative of the parental MDA-MB-231. STR-analysis was performed to validate each cell line. Both the MDA-MB-231 and MDA-MB-231^LG^ resulted in a 9/9 match for markers established by ATCC to confirm identity of cell lines corresponding to a 100% match by these criteria (Table 1). An additional 14 markers were also examined and demonstrated > 96% similarity with the only difference at marker D18S51 corresponding to a heterozygous versus homozygous result in parental MDA-MB-231 and MDA-MB-231^LG^ respectively.

### Increased membrane contact in MDA-MB-231^LG^

**Fig. 2:**
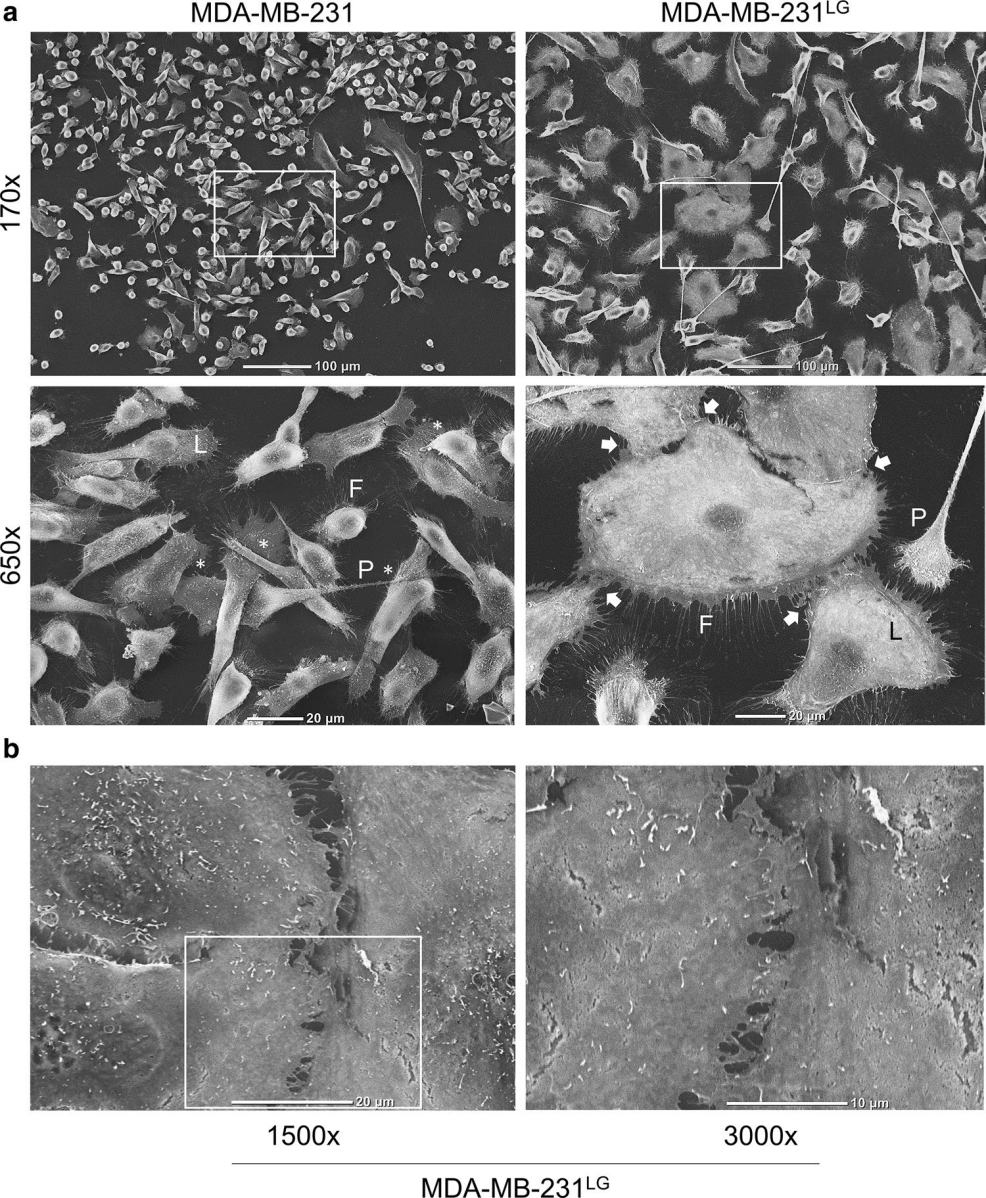
MDA-MB-231^LG^ exhibit increased membrane contact. **a** Analysis of MDA-MB-231 and MDA-MB-231^LG^ morphology and cell–cell interactions by scanning electron microscopy at indicated magnifications. *Indicates overlapping membranes; arrows indicate sites of cell–cell membrane contact. L: lamellipodia; F: filopodia; P: pseudopodia **b,** High magnification images of membrane interactions in MDA-MB-231^LG^

When observed by phase microscopy, MDA-MB-231^LG^ appeared to exhibit greater contact at the plasma membrane between cells. To examine this in more detail, we performed scanning electron microscopy of both the MDA-MB-231 and MDA-MB-231^LG^. Results demonstrated an expected mesenchymal phenotype in MDA-MB-231 showing an elongated appearance with overlapping membranes and minimal cell–cell junctions (Fig. 2a). In contrast, MDA-MB-231^LG^ clearly showed an increased ability to form cell–cell junctions with non-overlapping membrane connections along portions of their plasma membranes (Fig. 2a, b). While both cell lines exhibited lamellipodia and filopodia, long pseudopodia were noted in the MDA-MB-231^LG^ (Fig. 2a). High power analysis allowed for the observation of membrane contact between MDA-MB-231^LG^ cells (Fig. 2b).

**Fig. 3:**
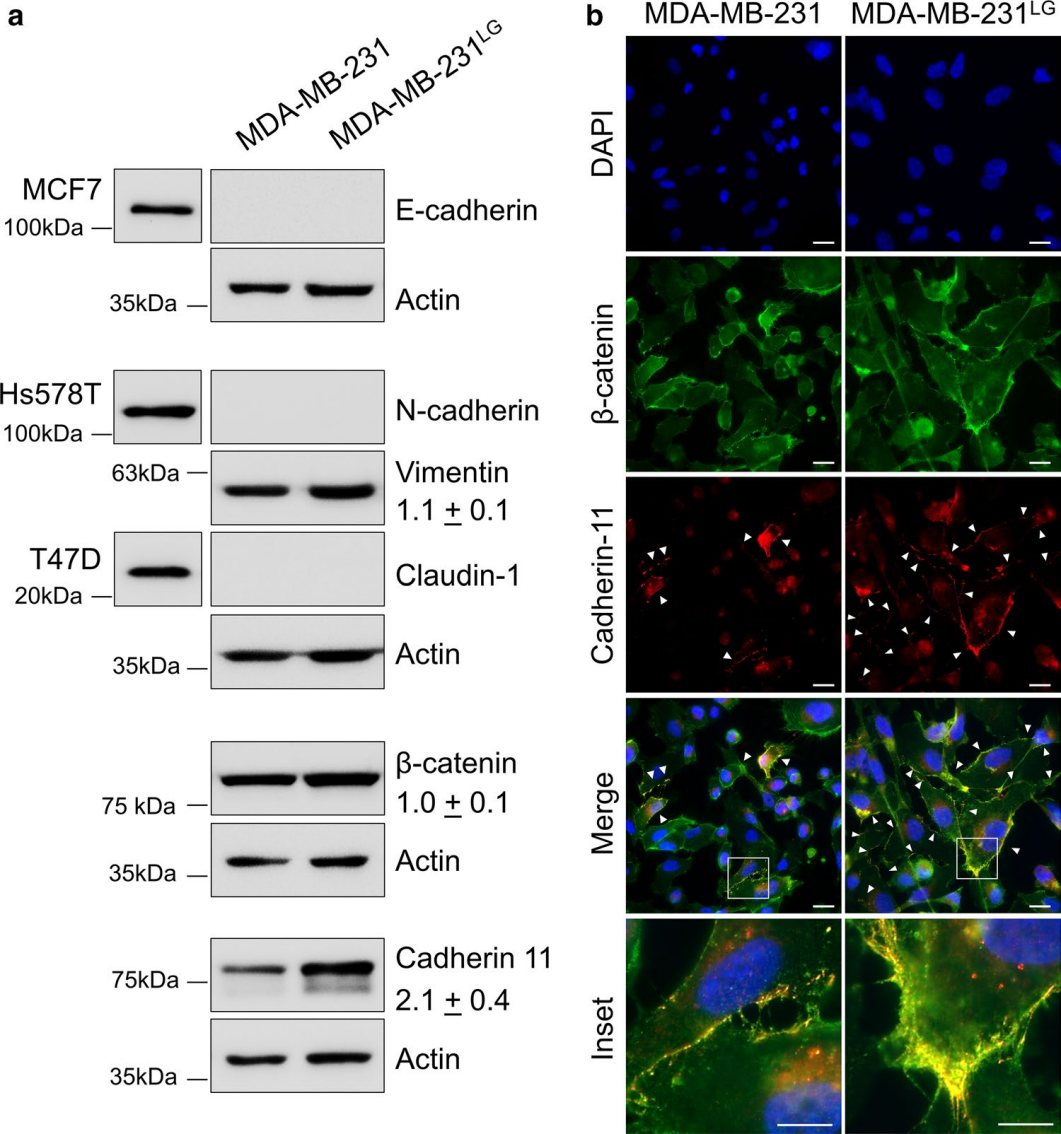
Cadherin-11 protein levels are increased in MDA-MB-231^LG^. **a** Representative western blot analyses for membrane and cytoskeletal EMT markers. All experiments performed three independent times. Detection of bands in positive control cell lines shown for E-cadherin, N-cadherin and claudin-1. Densitometry represents fold-change ± SD for vimentin, β-catenin and cadherin-11 in MDA-MB-231^LG^ compared to MDA-MB-231. β-actin used a loading control. **b** Representative immunofluorescence analysis of three independent experiments examining β-catenin and cadherin-11. Merged images demonstrate fluorescence overlap. DAPI, blue; β-catenin, green; cadherin-11, red. Scale bar: 20 µm; inset: 10 µm

Due to these observations, we examined markers of epithelial-to-mesenchymal transition (EMT) to determine if re-expression of epithelial markers may account for the increased cell–cell junctions. Epithelial-cadherin (E-cadherin, *CDH1*) and claudin-1 (*CLDN1*) were not detected in either MDA-MB-231 or MDA-MB-231^LG^ (Fig. 3a), indicating that upregulation of these markers was not responsible for the increased cell–cell attachment noted in the MDA-MB-231^LG^. N-cadherin (*CDH2*), a mesenchymal-associated cadherin was not detected in the MDA-MB-231 or the MDA-MB-231^LG^ and levels of the intermediate filament and mesenchymal marker vimentin (*VIM*) remained relatively unchanged (Fig. 3a). However, MDA-MB-231 have been shown to express cadherin-11 (*CDH11*) and analysis in the MDA-MB-231^LG^ demonstrated a significant increase in the levels of this attachment factor (Fig. 3a). When observed by immunofluorescence, strong membrane localization was observed throughout the cell population, particularly at sites of cell–cell contact (Fig. 3b). By comparison, cadherin-11 was observed intermittently throughout MDA-MB-231 populations. Levels of β-catenin (*CTNNB1*), a multi-function protein involved in both cellular signaling and cell–cell attachment, remained similar between the cell lines (Fig. 3a). However, increased fluorescence overlap of β-catenin with cadherin-11 at the plasma membrane was apparent in the MDA-MB-231^LG^ (Fig. 3b).

### Adaptation to low glucose increases connexin 43 and GJIC

**Fig. 4:**
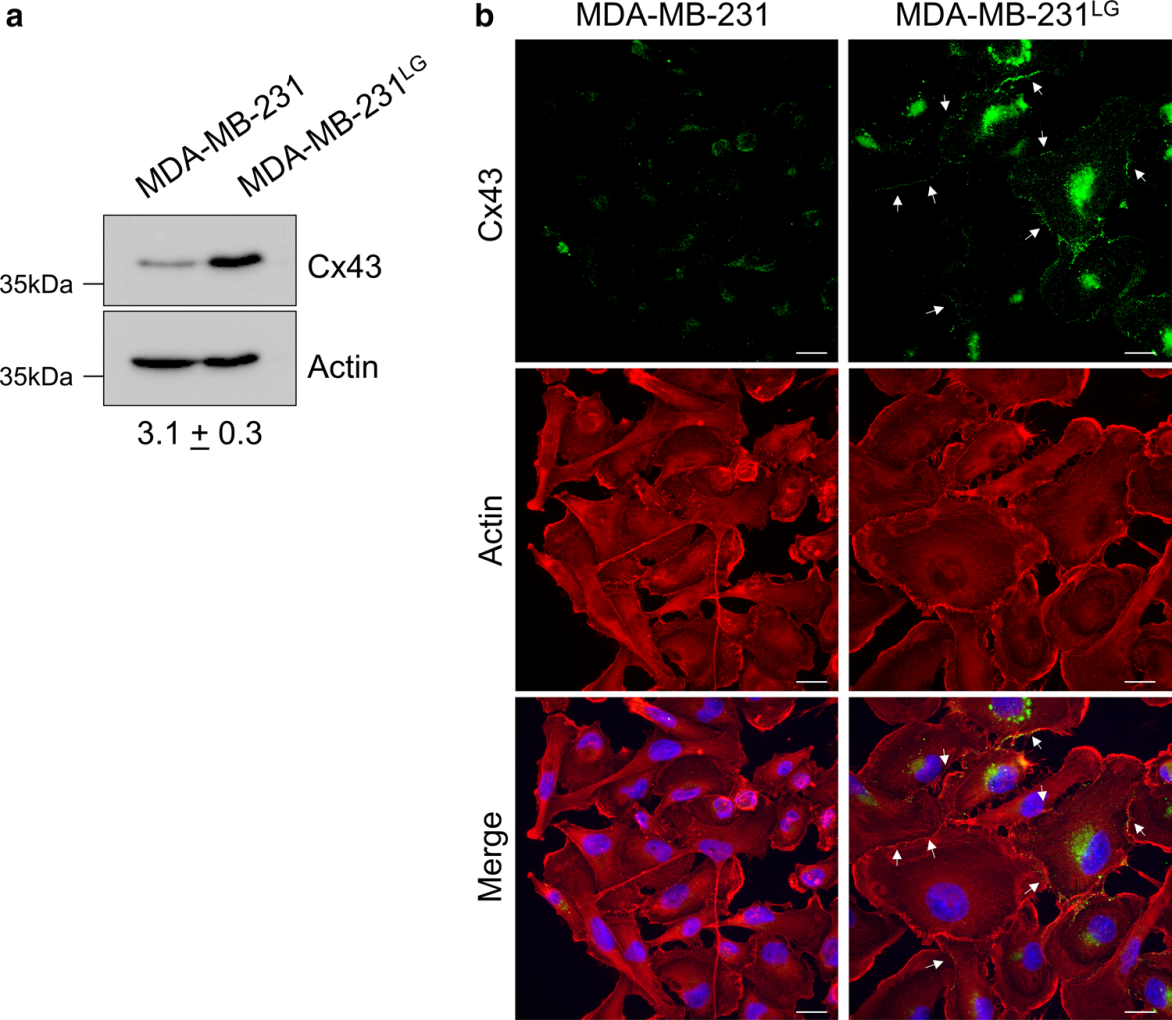
C×43 protein levels and membrane localization are increased in MDA-MB-231^LG^. **a** Representative western blot analysis of C×43 protein levels from whole cell lysates in three independent experiments. β-actin used as a loading control. Densitometry represents fold-change ± SD for C×43 in MDA-MB-231^LG^ compared to MDA-MB-231. **b** Representative immunofluorescence analysis of three independent experiments for C×43. DAPI: blue; C×43: green; actin: red. Scale bar: 20 µm. Additional fields shown in Supp. Figure 4

We next set out to determine if the metabolic adaptations in the MDA-MB-231^LG^ and alterations to membrane structure affected GJIC. We first examined protein levels of connexin 43 (*GJA1,* C×43), a major connexin protein expressed in breast tissue and found an increase in proteins levels of this connexin in the MDA-MB-231^LG^ (Fig. 4a). C×43 is subject to significant post-translational modification and higher molecular weight species of C×43 can be detected by western blot analysis (Supp. Fig. 3). However, in both the MDA-MB-231 and MDA-MB-231^LG^, we did not detect higher molecular weight species of C×43 (Fig. 4a).

**Fig. 5:**
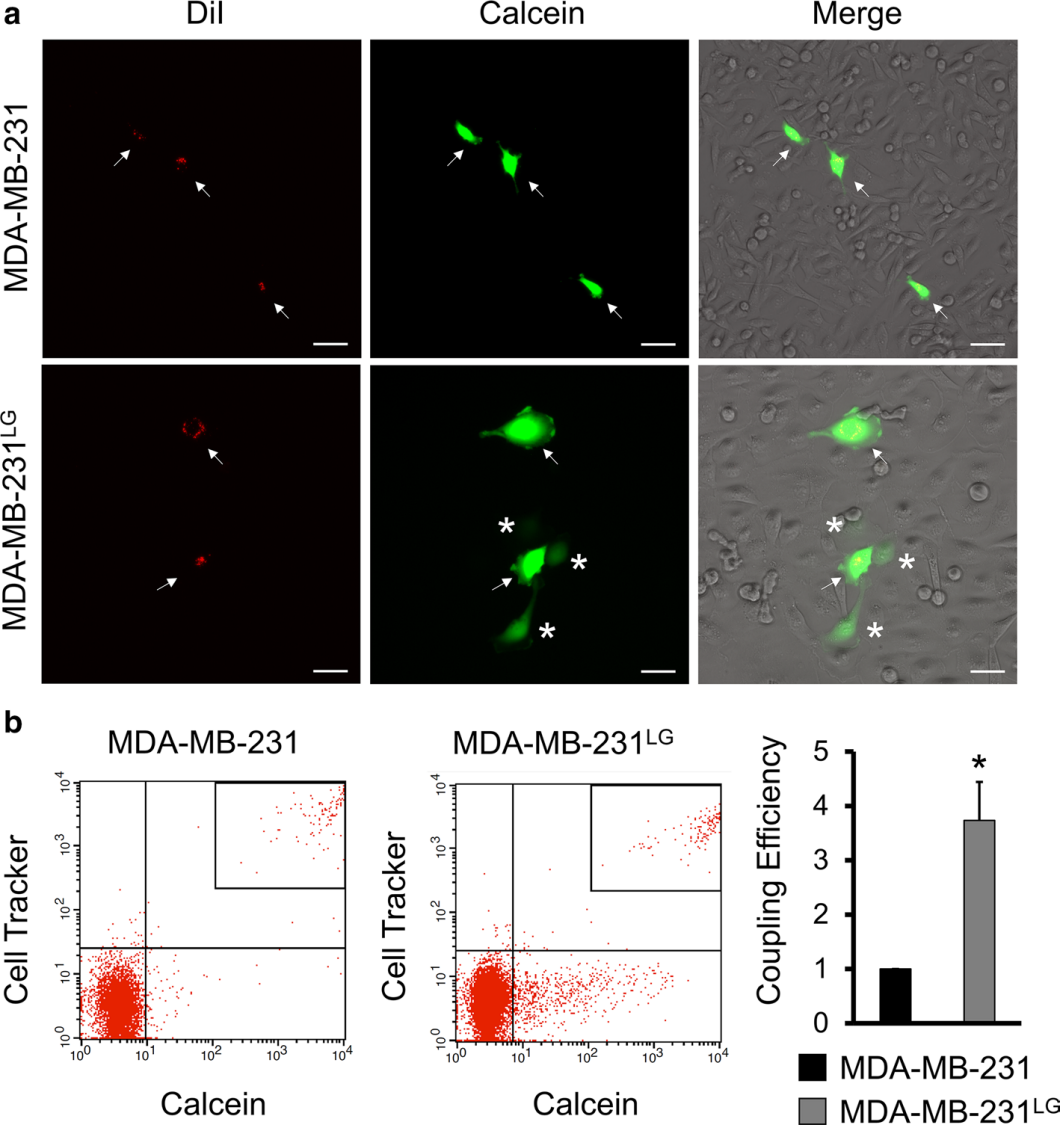
GJIC is increased in MDA-MB-231^LG^. **a** Double-label fluorescent dye transfer was used to observe GJIC. Transfer of calcein from CM-DiI labeled donor cells demonstrates active GJIC. Arrows indicate double-labeled donor cells; asterisk designate calcein positive acceptor cells. Representative fields shown. Calcein: green; CM-DiI: red. Scale bar: 50 µm. Data represent three independent experiments. **b** Flow cytometry quantification of coupling efficiency using Cell Tracker Deep Red to mark donor cells and calcein to measure GJIC. Upper right quadrant: double-labeled donor cells; lower left quadrant: non-labeled cells; bottom right quadrant: acceptor cells (calcein only). Experiments were performed in triplicate three independent times. Results were normalized to control and represented as fold change of MDA-MB-231^LG^ compared to MDA-MB-231. Data represent the mean ± SD. (*p < 0.05)

We then determined if membrane localization of C×43 was also affected in the MDA-MB-231^LG^. MDA-MB-231 showed minimal staining for C×43 that was predominantly peri-nuclear with little localization at the membrane (Fig. 4b). In contrast, MDA-MB-231^LG^ displayed a higher degree of C×43 localization at the plasma membrane, particularly at cell junctions, indicative of gap junction formation (Fig. 4b and Supp. Fig. 4). To determine if the increase in C×43 membrane localization corresponded to functional gap junctions, a double-label dye transfer technique was performed to assess GJIC with transfer of the fluorescent dye calcein indicating active GJIC. MDA-MB-231 exhibited minimal spread of calcein while a greater number of MDA-MB-231^LG^ were capable of transferring this dye to neighboring cells (Fig. 5a). This led to a measurable increase in GJIC when quantitatively assessed by flow cytometry (Fig. 5b).

### MDA-MB-231^LG^ exhibit increased invasive qualities

**Fig. 6:**
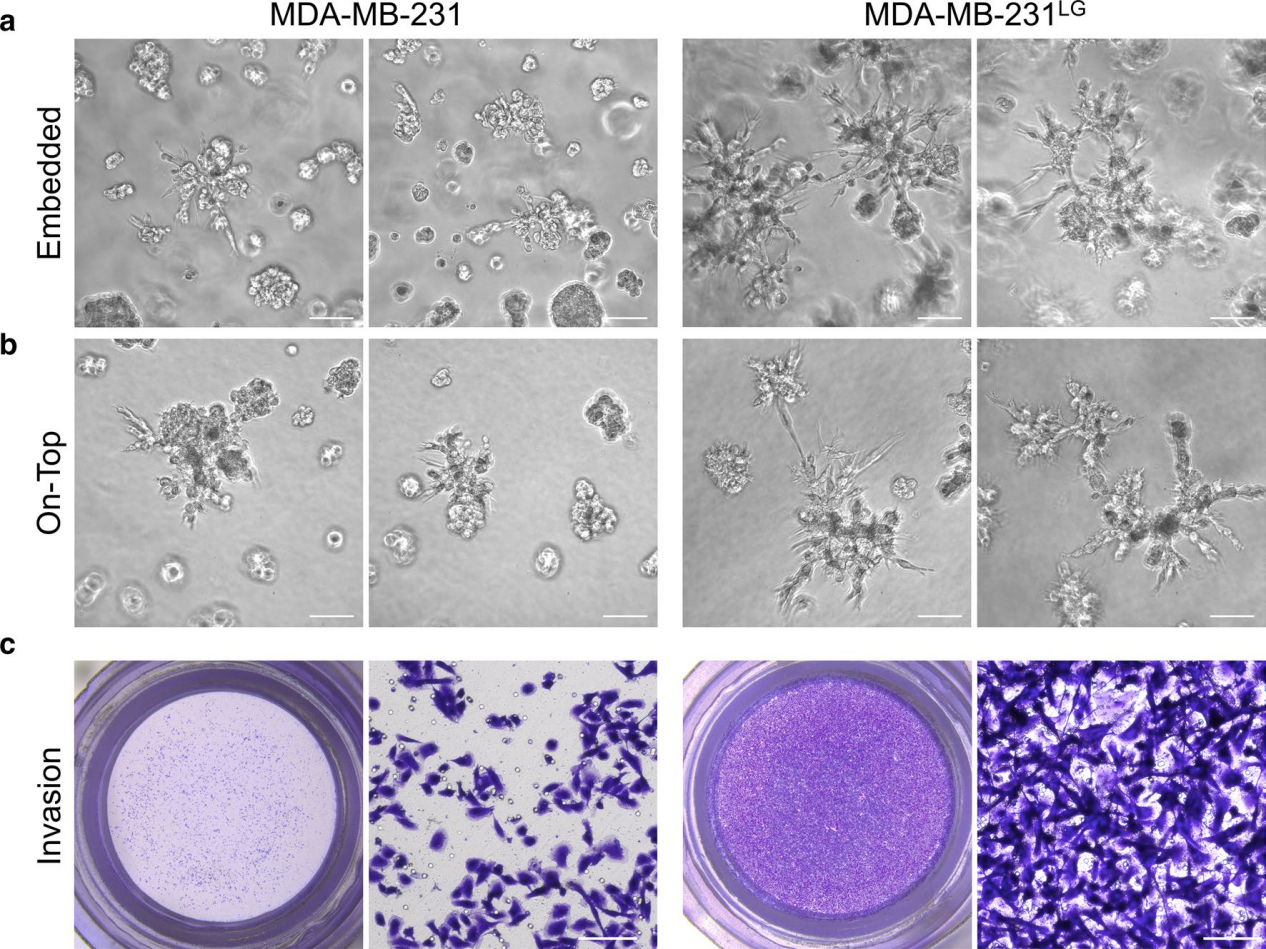
MDA-MB-231^LG^ display greater invasive qualities. **a** MDA-MB-231 and MDA-MB-231^LG^ were embedded in Matrigel membrane and allowed to grow for 6 days. Cells grew in colonies within the matrix and invasive protrusions of cells were indicative of invasive qualities. Two representative fields shown from three independent experiments. Scale bar: 100 µm. **b** Matrigel on-top procedure involved culture of cells on matrix overlaid with Matrigel and grown for 6 days. Two representative fields shown from three independent experiments. Scale bar: 100 µm. **c** Matrigel invasion chamber assays were performed in duplicate in three independent experiments for both cell lines with 24 h culture. MDA-MB-231 and MDA-MB-231^LG^ were grown in upper chambers in serum free media corresponding to each cell line. Lower chamber of inserts contained 5% FBS as a chemoattractant. Cells invading through the matrix and 8 µm pore inserts indicated invasion and were stained with crystal violet for visualization. Scale bar: 100 µm

Because changes to GJIC have been shown to affect the invasive capabilities of cancer cells, we sought to evaluate if this quality was also altered in the MDA-MB-231^LG^. We examined the morphologic appearance of these cells in three-dimensional culture utilizing Matrigel reconstituted basement membrane matrix. Cells were embedded within Matrigel, allowing for growth in three-dimension, a condition which more closely resembles cell growth in vivo and allows for observation of phenotypic characteristics. While both MDA-MB-231 and MDA-MB-231^LG^ formed spheroids, MDA-MB-231^LG^ exhibited greater stellate extensions at 6 days of culture (Fig. 6a). This was confirmed by use of a second assay in which cells were grown on top of a Matrigel matrix and overlaid with additional Matrigel (Fig. 6b). This procedure allows for more clear documentation of cellular morphologies and extensions and demonstrated a more invasive phenotype in MDA-MB-231^LG^. To determine if these changes to three-dimensional morphology correlated to increased invasive function, Matrigel invasion chamber assays were used to quantitatively assess the ability of each cell line to invade through a three-dimensional matrix and traverse a membrane containing 8 µm pores. Cells were seeded at the top of the matrix in serum-free media and FBS was used as a chemoattractant in the lower chamber of the assay. Invading cells were stained with crystal violet and MDA-MB-231^LG^ demonstrated a clear increase in the number of invading cells (Fig. 6c).

**Fig. 7:**
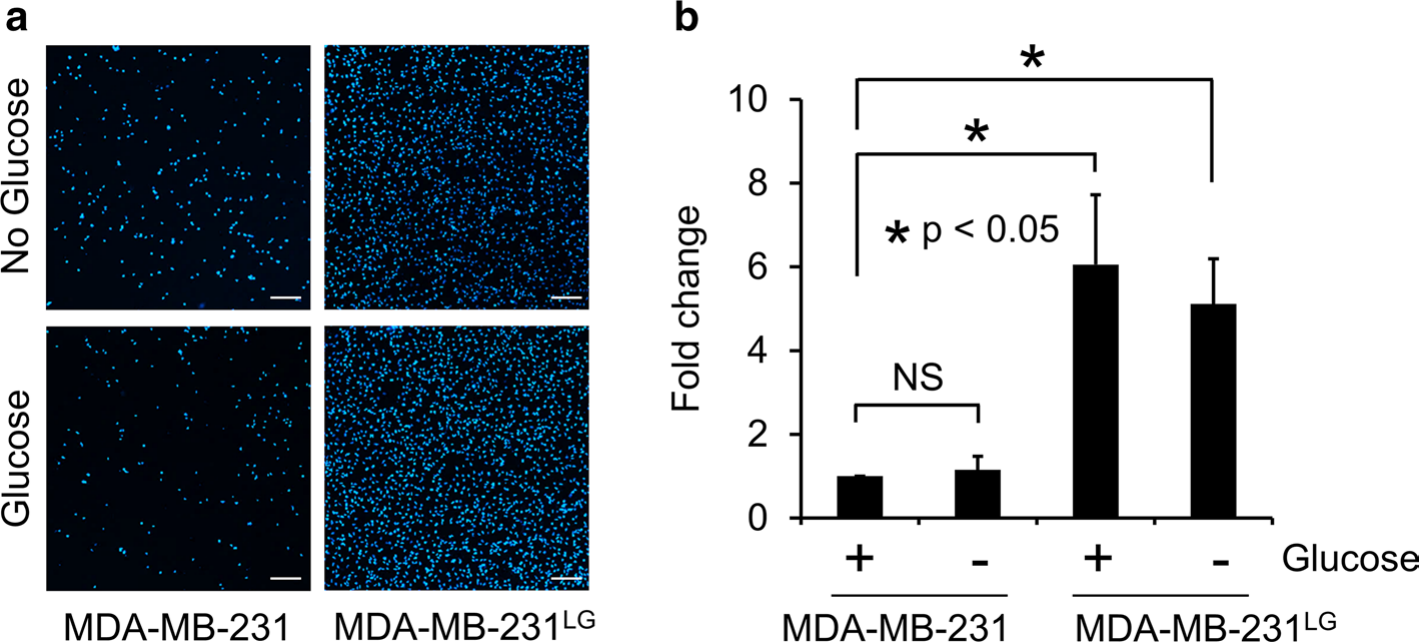
MDA-MB-231^LG^ maintain invasiveness in the presence of glucose. **a** Matrigel invasion chamber assays were performed in duplicate in three independent experiments in the presence or absence of glucose and invading cells stained with DAPI. Scale bar: 200 µm. **b** Quantification of results in **a**. Data represent the mean of three independent experiments normalized to MDA-MB-231 + glucose and shown as fold change ± SD. (*p < 0.05; NS, not significant)

To more quantitatively assess the invasive capacity of the MDA-MB-231^LG^ we adapted our invasion chamber conditions to fixation with DAPI and analyzed these images using fluorescence microscopy and computer-based software quantification. Additionally, to evaluate potential contributing effects of glucose availability on each cell line, we conducted these experiments in the presence or absence of glucose for 24 h. The MDA-MB-231^LG^ exhibited a significantly more invasive capacity in each condition compared to MDA-MB-231 (Fig. 7a, b). The overall results with MDA-MB-231^LG^ demonstrated significantly higher invasive qualities in each condition.

**Fig. 8:**
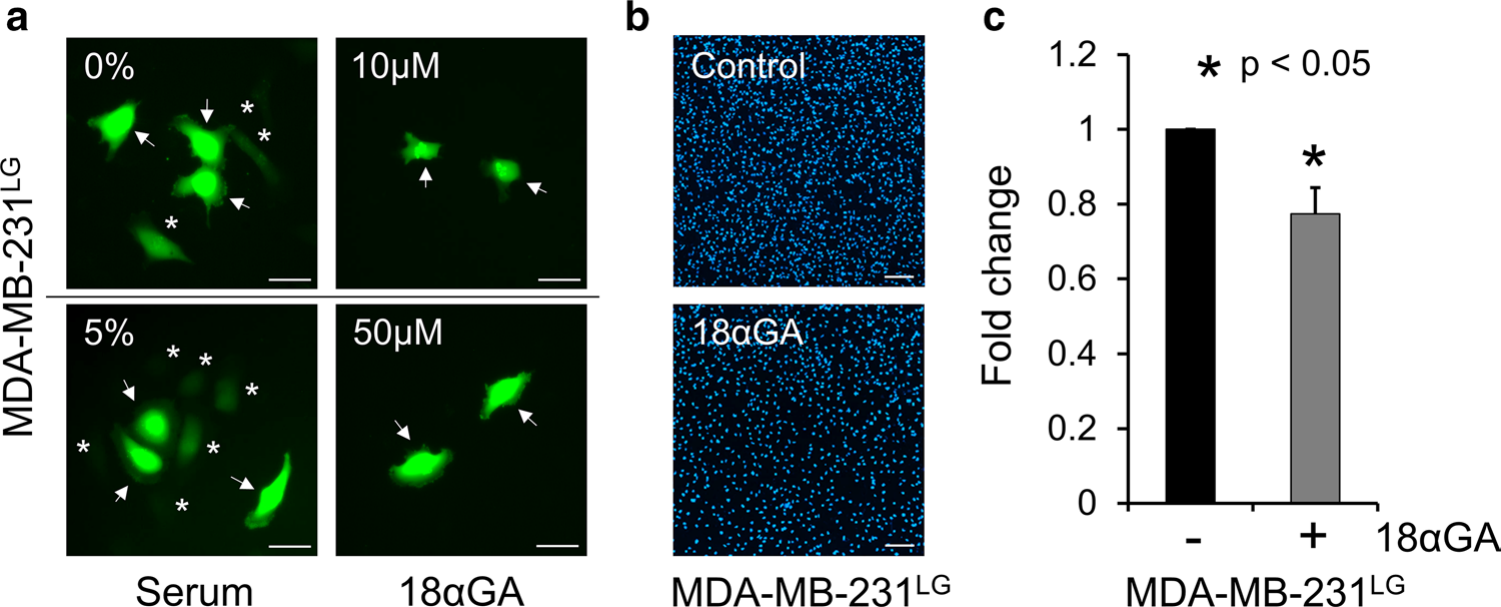
Inhibition of GJIC reduces invasion in MDA-MB-231^LG^. **a** Calcein dye transfer assays from three independent experiments performed in serum-free or 5% serum conditions ± indicated concentrations of 18αGA for 24 h. Arrows indicate donor cells, asterisks indicate acceptor cells. Scale bar: 50 µm. **b** Matrigel invasion chamber assays were performed in duplicate in three independent experiments with 5% FBS in the bottom chamber and serum-free media in the top chamber. Invading cells were stained with DAPI. Representative fields shown. Scale bar: 200 µm. **c** Quantification of results in **b**. Data was normalized to MDA-MB-231^LG^ in the absence of 18αGA and represented as mean ± SD. (*p < 0.05)

Finally, to examine a potential link between the increased GJIC in MDA-MB-231^LG^ and their invasive qualities, we used the gap junction un-coupling agent 18α-glycyrrhetinic acid (18αGA) to reduce GJIC in MDA-MB-231^LG^. To decrease the possibility of non-specific effects of 18αGA, we used the minimum concentration of 18αGA capable of inhibiting GJIC at 24 h for each condition in the Matrigel invasion chamber assay. This corresponded to 10 µM in serum-free conditions (upper chamber) and 50 µM in 5% FBS (lower chamber) (Fig. 8a).18αGA under these conditions caused a slight but significant reduction in Matrigel chamber invasion of MDA-MB-231^LG^ when compared to untreated conditions (Fig. 8b, c).

## Discussion

During cancer progression, breast cancer cells exhibit a multitude of responses to the extracellular milieu in which they exist. Among these alterations are changes in GJIC and adaptation to fluctuating metabolic conditions. A better understanding of how these two processes affect each other, and how this contributes to the cancer cell phenotype, will provide important information about this relationship. In this report, we describe the generation of a metabolic variant of the MDA-MB-231 parental cell line adapted for growth in glucose-limiting conditions. These cells maintained a proliferative capacity and high viability despite reduced utilization of this carbon source. The increased levels of autophagy that we observed in the MDA-MB-231^LG^ are likely a contributing factor to their survival, yet not high enough to induce autophagic (type II) cell death. We believe that generation of the MDA-MB-231^LG^ represent a metabolic adaptation to these growth parameters due to their significantly altered morphology as well as the molecular and functional changes we have characterized. However, persistent subpopulations within the MDA-MB-231 have been reported (Louie et al. 2010; Wang et al. 2015; Amaro et al. 2016; Morata-Tarifa et al. 2016). Therefore, the possibility that the MDA-MB-231^LG^ represent isolation of an existing variant through the selective metabolic pressure described in our experiments remains possible. Although these cells displayed significant differences in appearance and function compared to the parental MDA-MB-231, STR analysis validated these cells a true derivative. Thus, we evaluated the characteristics of the MDA-MB-231^LG^ on GJIC and invasion to determine how this metabolic adaptation affected these qualities.

The MDA-MB-231 cell line was originally isolated from a pleural effusion of a patient with an intraductal carcinoma and displayed rounded to spindle-like morphology following establishment in culture (Cailleau et al. 1974). Cells were isolated post-chemotherapeutic treatment and displayed significant aneuploidy. Molecular characterization has revealed a triple-negative profile with mutations in numerous genes including *TP53*, *BRAF* and *KRAS* (Hollestelle et al. 2010). The MDA-MB-231 exhibit a phenotype consistent with EMT and express mesenchymal markers such as the intermediate filament vimentin and lack of epithelial proteins such as E-cadherin (Nieman et al. 1999; Pishvaian et al. 1999; Li et al. 2011; Liu et al. 2015). MDA-MB-231 also fall in the category of “claudin-low” due to their decreased expression of tight junction proteins such as claudin-1 (Majer et al. 2016; Dias et al. 2017; Chiang et al. 2019; Mattern et al. 2019). Collectively, this promotes a decrease in cell–cell attachment and increase in cellular motility. Because the MDA-MB-231^LG^ exhibited stronger cell–cell contacts and altered morphology, we explored if these cells underwent a reversal of this process known as mesenchymal-to-epithelial transition (MET) by analysis of cytoskeletal and membrane EMT markers (Chao et al. 2010; Liu et al. 2016). No upregulation of E-cadherin or claudin-1 was observed. Additionally, N-cadherin, a mesenchymal-associated cadherin, was not detected in the MDA-MB-231 (consistent with previous reports (Hazan et al. 1997; Nieman et al. 1999; Wang et al. 2002; Shankar and Nabi 2015) and could not be detected in the MDA-MB-231^LG^. However, MDA-MB-231 express cadherin-11 (Nieman et al. 1999; Wang et al. 2002; Li et al. 2011; Satriyo et al. 2019). Cadherin-11, like other cadherins, promotes cell–cell contact through homomeric extracellular interactions that help to link cytoskeletal proteins on the cytoplasmic side. Cadherin-11 is upregulated in invasive breast cancer (Assefnia et al. 2014; Pohlodek et al. 2016). Moreover, inhibition of cadherin-11 in the MDA-MB-231 decreased migration, growth in soft agar, stem cell marker expression, and in vivo tumor growth (Assefnia et al. 2014; Satriyo et al. 2019). The observation of higher cadherin-11 levels in the MDA-MB-231^LG^ may contribute to the increase in cell–cell contact in these cells as it exhibited a membrane localized pattern, particularly at sites of cell–cell interaction. Interestingly, we observed a corresponding increase of cadherin-11 at sites of β-catenin near the plasma membrane. β-catenin exhibits multiple subcellular localizations within the nucleus, cytosol and plasma membrane corresponding to different functions and activity. β-catenin at the plasma membrane is involved in linking adherens junctions to cytoskeletal proteins such as actin and can contribute to cytoskeletal reorganization dynamics. The markers examined here represent only a small fraction of the many EMT, membrane and cytoskeletal proteins altered in cancer and further characterization is required to confirm a relationship. Nonetheless, these results suggest a potential relationship to the morphologic and membrane changes seen in the MDA-MB-231^LG^.

Since membrane contact is a fundamental aspect of GJIC, we explored if the plasma membrane interactions observed in the MDA-MB-231^LG^ correlated with the formation of gap junctions. C×43 is highly expressed in breast tissue and one of the most well studied connexins altered during cancer progression. Similar to previous reports, we observed that parental MDA-MB-231 expressed low levels of C×43 with the absence of plasma membrane localization and GJIC in these cells (Qin et al. 2001; 2003, Jiang et al. 2017). Remarkably, C×43 protein was elevated in MDA-MB-231^LG^. We observed predominantly lower molecular weight forms of C×43 in each cell line similar to previous reports in MDA-MB-231 (Qin et al. 2002; Talhouk et al. 2013; Ming et al. 2015). However, the increase in C×43 indicated a potential adaptive response related to glucose levels and C×43 expression. Conversely, a recent report demonstrated an inverse relationship between high-glucose levels and C×43 expression in osteocyte-like cells (Yang et al. 2020). Augmented connexin protein levels alone are not capable of increasing GJIC when trafficking and membrane transport of connexin proteins remains dysregulated (Qin et al. 2001, 2003). Thus, we evaluated C×43 subcellular localization in MDA-MB-231^LG^ cells and found an increase in the presence of this connexin at the membrane, indicative of connexon formation and membrane incorporation. To demonstrate function, we documented the ability of MDA-MB-231^LG^ to transfer calcein to neighboring cells and noted an increase in GJIC. An important observation was that not all MDA-MB-231^LG^ were coupled via gap junctions, and only a portion of the cells exhibited spread. However, the results described in this report regarding C×43 and GJIC are noteworthy because they developed as a consequence of the metabolic response to growth in glucose-limiting conditions without the influence of exogenous connexin expression or the use of pathway inhibitors or activators. Since this is a metabolic circumstance frequently overcome by cancer cells during tumor growth, it provides insight to a potential reprogramming response involving connexins and GJIC that may affect their ability to survive and adapt. Because the connexin family consists of 21 genes, some of which have been shown to be upregulated in different types of cancers and in response to different stimuli, we cannot exclude the influence of other connexins in the MDA-MB-231^LG^.

Because cancer cell invasion is a key initial feature of the metastatic process, and because GJIC has been shown to affect this process, we examined if these collective changes affected this aggressive quality. We used Matrigel to evaluate multiple three-dimensional in vitro growth models to characterize cellular invasion. Despite greater membrane contact, increased connexin expression and higher GJIC, all features more similar to normal epithelial cells, the MDA-MB-231^LG^ demonstrated significantly higher invasive qualities in both the presence and absence of glucose, indicating intrinsic qualities in these cells. Previous studies which directly modulated expression of C×43 in the MDA-MB-231 cell line have shown reduction in proliferation, invasive characteristics in three-dimensional culture and an increased state of differentiation in response to C×43 expression while an inverse relationship was observed following reduction of C×43 (Qin et al. 2002; Shao et al. 2005; McLachlan et al. 2006; Talhouk et al. 2013). Some of these effects were attributed to gap junction independent functions of C×43. Other studies using this cell line have demonstrated both suppression and facilitation of aggressive and metastatic qualities in response to C×43 expression (Li et al. 2008; Fu et al. 2015; Lin et al. 2016; Kazan et al. 2019). Because this study examined the effects on C×43 expression as a result of changes to glucose metabolism, our results are therefore set amongst the background of this metabolic adaptation. Collectively, these studies emphasize the complexities related to the role of C×43 in cancer cells that are dependent upon experimental approach and context, highlighting the intricacies related to the study of GJIC and cancer.

To examine if the increase in invasiveness and GJIC in the MDA-MB-231^LG^ were connected, we evaluated the invasive qualities of these cells during the inhibition of GJIC. We utilized 18α-glycerrehetinic acid (18αGA), a nonselective gap junction inhibitor which uncouples GJIC (Davidson et al. 1986; Salameh and Dhein 2005).The reduced invasive capacity of these cells in the presence of 18αGA indicated a potential link between these two attributes. The fact that not all MDA-MB-231^LG^ exhibited GJIC must be considered when examining the change in invasiveness during 18αGA treatment. The effects on invasion under these conditions may have been more prominent if the population was more uniformly coupled by GJIC. Thus, careful delineation of these potential relationships will be of importance to our understanding of connexin function in response to metabolic regulation. The results of this study shed light on phenotypic changes that occur in response to nutrient withdrawal and further support a role for cellular metabolism on the regulation of connexins and gap junctional activity in this context.

## Electronic supplementary material

Below is the link to the electronic supplementary material.

Validation of cellular viability assay by flow cytometry in MDA-MB-231. a, Caspase 3/7 positive control conditions were prepared by treating cells with 1µM staurosporine for 18 h. b, SYTOX positive controls were prepared by heat killing cells at 65 ^o^C for 5 minutes. (TIFF 1277 kb)

Demonstration of gap junction assay using calcein and CM-DiI. Hs578T exhibit high levels of GJIC. a, Donor cells were loaded with CM-DiI and calcein and co-cultured with acceptor cells for 6 h. Spread of calcein from double-labeled donor cells indicates activity of GJIC. Calcein: green; CM-DiI: red. Scale bar: 50 µm; n = 3. b, Quantification of gap junction assay by flow cytometry using Cell Tracker Red to mark donor cells. Double-labeled donor cell populations appear in upper-right quadrant when calcein is plotted on the x-axis and Cell Tracker Red plotted on the y-axis. Non-labeled cells appear in lower-left quadrant indicating absence of both dyes. Following 6 h co-culture of these populations at a ratio of 1:20 donor cell/acceptor cell for 6 h, calcein-only positive acceptor populations appear in the lower-right quadrant. Coupling efficiency is calculated as the number of acceptor cells divided by the number of donor cells in the experiment ± SD. (TIFF 9174 kb)

Lysates of HEK293T cells with empty vector control and Cx43 overexpression were analyzed by western blot analysis. Arrows indicate multiple molecular weight bands for Cx43. GAPDH was used as a loading control. (TIFF 1486 kb)

Additional fields of Cx43 immunofluorescence in MDA-MB-231 (a) and MDA-MB-231^LG^ (b) as described in Figure 4. DAPI: blue; Cx43: green; actin: red. Scale bar represents 20 µm. (TIFF 30503 kb)

STR analysis of Hs578T, MCF7 and T47D. Asterisk indicate 9 markers defined by ATCC criteria for 100% match. (TIFF 3004 kb)

## Abbreviations

C×43: Connexin 43
GJIC: Gap junctional intercellular communication

## References

[CR1] Aasen T, Mesnil M, Naus CC (2016). Gap junctions and cancer: communicating for 50 years. Nat Rev Cancer.

[CR2] Amaro A, Angelini G, Mirisola V (2016). A highly invasive subpopulation of MDA-MB-231 breast cancer cells shows accelerated growth, differential chemoresistance, features of apocrine tumors and reduced tumorigenicity in vivo. Oncotarget.

[CR3] Assefnia S, Dakshanamurthy S, Guidry Auvil JM (2014). Cadherin-11 in poor prognosis malignancies and rheumatoid arthritis: common target, common therapies. Oncotarget.

[CR4] Bates DC, Sin WC, Aftab Q (2007). Connexin43 enhances glioma invasion by a mechanism involving the carboxy terminus. Glia.

[CR5] Cailleau R, Young R, Olive M (1974). Breast tumor cell lines from pleural effusions. J Natl Cancer Inst.

[CR6] Chao YL, Shepard CR, Wells A (2010). Breast carcinoma cells re-express E-cadherin during mesenchymal to epithelial reverting transition. Mol Cancer.

[CR7] Chen Q, Boire A, Jin X (2016). Carcinoma-astrocyte gap junctions promote brain metastasis by cGAMP transfer. Nature.

[CR8] Chiang SK, Chang WC, Chen SE (2019). DOCK1 regulates growth and motility through the RRP1B-claudin-1 pathway in claudin-low breast cancer cells. Cancers (Basel).

[CR9] Contreras JE, Sanchez HA, Eugenin EA (2002). Metabolic inhibition induces opening of unapposed connexin 43 gap junction hemichannels and reduces gap junctional communication in cortical astrocytes in culture. Proc Natl Acad Sci.

[CR10] Czyz J, Irmer U, Schulz G (2000). Gap-junctional coupling measured by flow cytometry. Exp Cell Res.

[CR11] Davidson JS, Baumgarten IM, Harley EH (1986). Reversible inhibition of intercellular junctional communication by glycyrrhetinic acid. Biochem Biophys Res Commun.

[CR12] DeBerardinis RJ, Chandel NS (2016). Fundamentals of cancer metabolism. Sci Adv.

[CR13] Dias K, Dvorkin-Gheva A, Hallett RM (2017). Claudin-low breast cancer; clinical & pathological characteristics. PLoS ONE.

[CR14] Dovmark TH, Saccomano M, Hulikova A (2017). Connexin-43 channels are a pathway for discharging lactate from glycolytic pancreatic ductal adenocarcinoma cells. Oncogene.

[CR15] Dovmark TH, Hulikova A, Niederer SA (2018). Normoxic cells remotely regulate the acid-base balance of cells at the hypoxic core of connexin-coupled tumor growths. FASEB J.

[CR16] el-Sabban ME, Pauli BU (1994). Adhesion-mediated gap junctional communication between lung-metastatatic cancer cells and endothelium. Invasion Metastasis.

[CR17] Fu Y, Shao ZM, He QZ (2015). Hsa-miR-206 represses the proliferation and invasion of breast cancer cells by targeting C×43. Eur Rev Med Pharmacol Sci.

[CR18] Garcia-Jimenez C, Goding CR (2019). Starvation and pseudo-starvation as drivers of cancer metastasis through translation reprogramming. Cell Metab.

[CR19] Ghosh S, Kumar A, Tripathi RP (2014). Connexin-43 regulates p38-mediated cell migration and invasion induced selectively in tumour cells by low doses of gamma-radiation in an ERK-1/2-independent manner. Carcinogenesis.

[CR20] Gillies RJ, Schornack PA, Secomb TW (1999). Causes and effects of heterogeneous perfusion in tumors. Neoplasia.

[CR21] Goldberg GS, Bechberger JF, Naus CC (1995). A pre-loading method of evaluating gap junctional communication by fluorescent dye transfer. Biotechniques.

[CR22] Hazan RB, Kang L, Whooley BP (1997). N-cadherin promotes adhesion between invasive breast cancer cells and the stroma. Cell Adhes Commun.

[CR23] Hollestelle A, Nagel JH, Smid M (2010). Distinct gene mutation profiles among luminal-type and basal-type breast cancer cell lines. Breast Cancer Res Treat.

[CR24] Hong X, Sin WC, Harris AL (2015). Gap junctions modulate glioma invasion by direct transfer of microRNA. Oncotarget.

[CR25] Jiang G, Dong S, Yu M (2017). Influence of gap junction intercellular communication composed of connexin 43 on the antineoplastic effect of adriamycin in breast cancer cells. Oncol Lett.

[CR26] Kazan JM, El-Saghir J, Saliba J (2019). C×43 expression correlates with breast cancer metastasis in MDA-MB-231 cells in vitro, in a mouse xenograft model and in human breast cancer tissues. Cancers (Basel).

[CR27] King TJ, Lampe PD (2004). The gap junction protein connexin32 is a mouse lung tumor suppressor. Cancer Res.

[CR28] Lamiche C, Clarhaut J, Strale PO (2012). The gap junction protein C×43 is involved in the bone-targeted metastatic behaviour of human prostate cancer cells. Clin Exp Metastasis.

[CR29] Li Z, Zhou Z, Donahue HJ (2008). Alterations in C×43 and OB-cadherin affect breast cancer cell metastatic potential. Clin Exp Metastasis.

[CR30] Li Y, Guo Z, Chen H (2011). HOXC8-dependent cadherin 11 expression facilitates breast cancer cell migration through trio and rac. Genes Cancer.

[CR31] Lin ZJ, Ming J, Yang L (2016). Mechanism of regulatory effect of MicroRNA-206 on Connexin 43 in distant metastasis of breast cancer. Chin Med J (Engl).

[CR32] Liu CY, Lin HH, Tang MJ (2015). Vimentin contributes to epithelial-mesenchymal transition cancer cell mechanics by mediating cytoskeletal organization and focal adhesion maturation. Oncotarget.

[CR33] Liu F, Gu LN, Shan BE (2016). Biomarkers for EMT and MET in breast cancer: an update. Oncol Lett.

[CR34] Louie E, Nik S, Chen JS (2010). Identification of a stem-like cell population by exposing metastatic breast cancer cell lines to repetitive cycles of hypoxia and reoxygenation. Breast Cancer Res.

[CR35] Majer A, Blanchard AA, Medina S (2016). Claudin 1 expression levels affect miRNA dynamics in human basal-like breast cancer cells. DNA Cell Biol.

[CR36] Mattern J, Roghi CS, Hurtz M (2019). ADAM15 mediates upregulation of Claudin-1 expression in breast cancer cells. Sci Rep.

[CR37] McLachlan E, Shao Q, Wang HL (2006). Connexins act as tumor suppressors in three-dimensional mammary cell organoids by regulating differentiation and angiogenesis. Cancer Res.

[CR38] Mehta PP, Hotz-Wagenblatt A, Rose B (1991). Incorporation of the gene for a cell-cell channel protein into transformed cells leads to normalization of growth. J Membr Biol.

[CR39] Ming J, Zhou Y, Du J (2015). miR-381 suppresses C/EBPalpha-dependent C×43 expression in breast cancer cells. Biosci Rep.

[CR40] Morata-Tarifa C, Jimenez G, Garcia MA (2016). Low adherent cancer cell subpopulations are enriched in tumorigenic and metastatic epithelial-to-mesenchymal transition-induced cancer stem-like cells. Sci Rep.

[CR41] Nielsen MS, Axelsen LN, Sorgen PL (2012). Gap junctions. Compr Physiol.

[CR42] Nieman MT, Prudoff RS, Johnson KR (1999). N-cadherin promotes motility in human breast cancer cells regardless of their E-cadherin expression. J Cell Biol.

[CR43] Ogawa K, Pitchakarn P, Suzuki S (2012). Silencing of connexin 43 suppresses invasion, migration and lung metastasis of rat hepatocellular carcinoma cells. Cancer Sci.

[CR44] Pavlova NN, Thompson CB (2016). The emerging hallmarks of cancer metabolism. Cell Metab.

[CR45] Pishvaian MJ, Feltes CM, Thompson P (1999). Cadherin-11 is expressed in invasive breast cancer cell lines. Cancer Res.

[CR46] Pohlodek K, Tan YY, Singer CF (2016). Cadherin-11 expression is upregulated in invasive human breast cancer. Oncol Lett.

[CR47] Qin H, Shao Q, Belliveau DJ (2001). Aggregated DsRed-tagged C×43 and over-expressed C×43 are targeted to lysosomes in human breast cancer cells. Cell Commun Adhes.

[CR48] Qin H, Shao Q, Curtis H (2002). Retroviral delivery of connexin genes to human breast tumor cells inhibits in vivo tumor growth by a mechanism that is independent of significant gap junctional intercellular communication. J Biol Chem.

[CR49] Qin H, Shao Q, Igdoura SA (2003). Lysosomal and proteasomal degradation play distinct roles in the life cycle of C×43 in gap junctional intercellular communication-deficient and -competent breast tumor cells. J Biol Chem.

[CR50] Salameh A, Dhein S (2005). Pharmacology of gap junctions. New pharmacological targets for treatment of arrhythmia, seizure and cancer?. Biochim Biophys Acta.

[CR51] Satriyo PB, Bamodu OA, Chen JH (2019). Cadherin 11 inhibition downregulates beta-catenin, deactivates the canonical WNT signalling pathway and suppresses the cancer stem cell-like phenotype of triple negative breast cancer. J Clin Med.

[CR52] Shankar J, Nabi IR (2015). Actin cytoskeleton regulation of epithelial mesenchymal transition in metastatic cancer cells. PLoS ONE.

[CR53] Shao Q, Wang H, McLachlan E (2005). Down-regulation of C×43 by retroviral delivery of small interfering RNA promotes an aggressive breast cancer cell phenotype. Cancer Res.

[CR54] Simoes RV, Serganova IS, Kruchevsky N (2015). Metabolic plasticity of metastatic breast cancer cells: adaptation to changes in the microenvironment. Neoplasia.

[CR55] Talhouk RS, Fares MB, Rahme GJ (2013). Context dependent reversion of tumor phenotype by connexin-43 expression in MDA-MB231 cells and MCF-7 cells: role of beta-catenin/connexin43 association. Exp Cell Res.

[CR56] Tang B, Peng ZH, Yu PW (2013). Aberrant expression of C×43 is associated with the peritoneal metastasis of gastric cancer and C×43-mediated gap junction enhances gastric cancer cell diapedesis from peritoneal mesothelium. PLoS ONE.

[CR57] Vaupel P, Kallinowski F, Okunieff P (1989). Blood flow, oxygen and nutrient supply, and metabolic microenvironment of human tumors: a review. Cancer Res.

[CR58] Wang F, Hansen RK, Radisky D (2002). Phenotypic reversion or death of cancer cells by altering signaling pathways in three-dimensional contexts. J Natl Cancer Inst.

[CR59] Wang WK, Chen MC, Leong HF (2014). Connexin 43 suppresses tumor angiogenesis by down-regulation of vascular endothelial growth factor via hypoxic-induced factor-1alpha. Int J Mol Sci.

[CR60] Wang X, Liu Y, Zhou K (2015). Isolation and characterization of CD105 +/CD90 + subpopulation in breast cancer MDA-MB-231 cell line. Int J Clin Exp Pathol.

[CR61] Yang L, Zhou G, Li M (2020). High glucose downregulates Connexin 43 expression and its gap junction and hemichannel function in osteocyte-like MLO-Y4 cells through activation of the p38MAPK/ERK signal pathway. Diabetes Metab Syndr Obes.

[CR62] Zibara K, Awada Z, Dib L (2015). Anti-angiogenesis therapy and gap junction inhibition reduce MDA-MB-231 breast cancer cell invasion and metastasis in vitro and in vivo. Sci Rep.

