# Supplementary figures and images for: Glucose-limiting conditions induce an invasive population of MDA-MB-231 breast cancer cells with increased connexin 43 expression and membrane localization
Source: J Cell Commun Signal. 2021 Feb 16;15(2):223–36. doi: 10.1007/s12079-020-00601-3 (PMC7991056; doi:10.1007/s12079-020-00601-3)

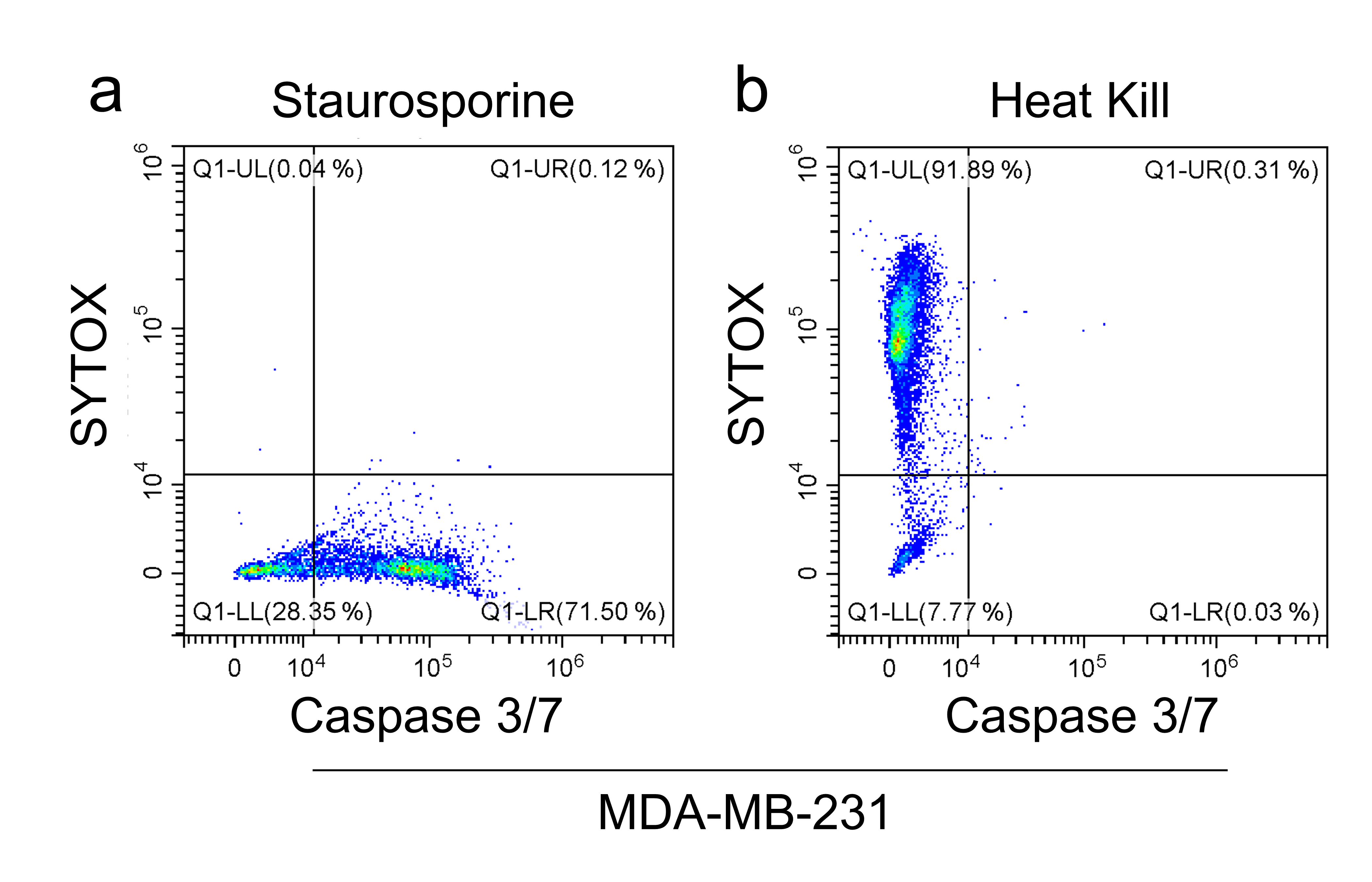

Supplement: Supplementary file 1 — Validation of cellular viability assay by flow cytometry in MDA-MB-231. a, Caspase 3/7 positive control conditions were prepared by treating cells with 1µM staurosporine for 18 h. b, SYTOX positive controls were prepared by heat killing cells at 65 oC for 5 minutes. (TIFF 1277 kb) [file 12079_2020_601_MOESM1_ESM.tif]

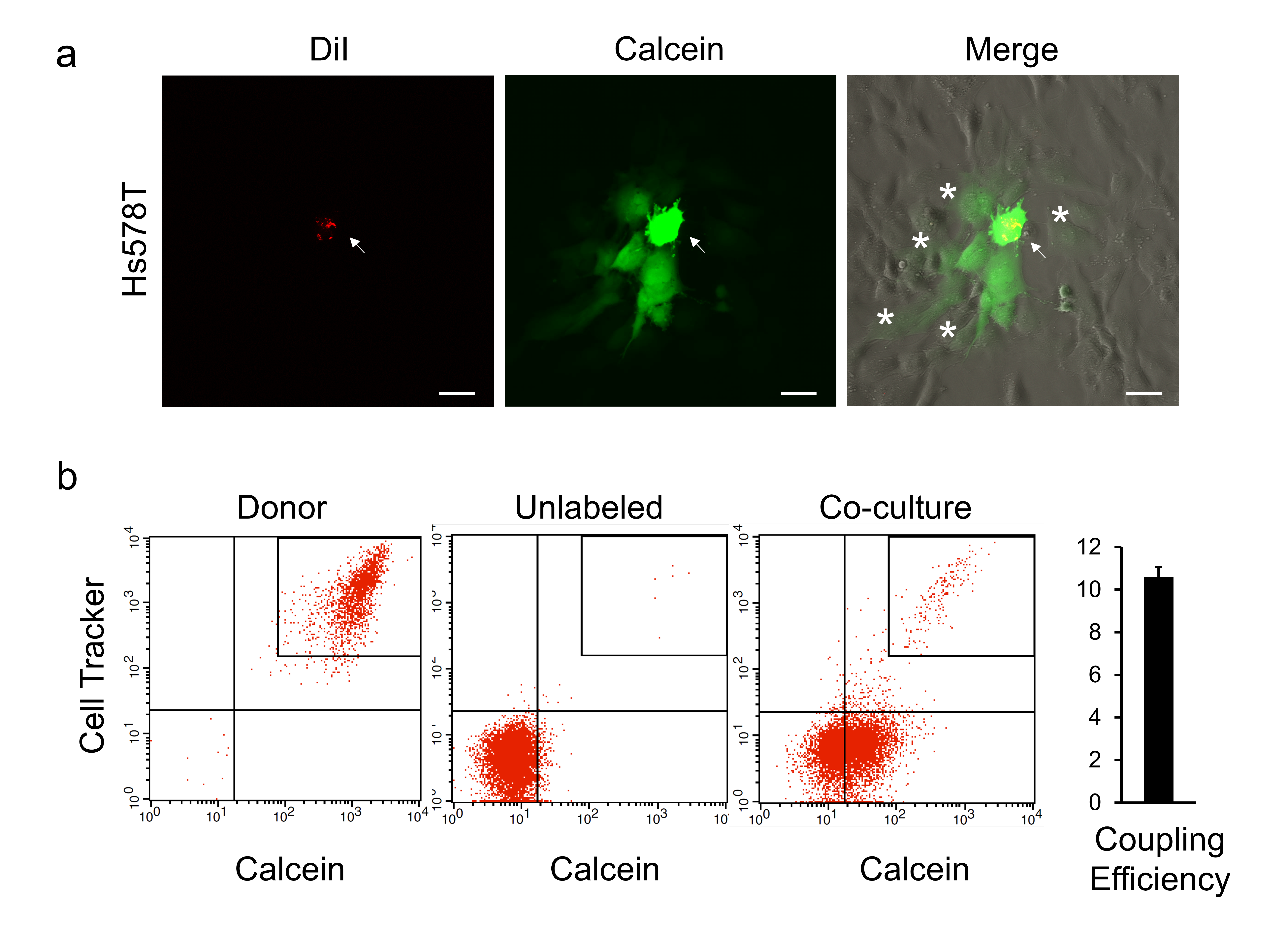

Supplement: Supplementary file 2 — Demonstration of gap junction assay using calcein and CM-DiI. Hs578T exhibit high levels of GJIC. a, Donor cells were loaded with CM-DiI and calcein and co-cultured with acceptor cells for 6 h. Spread of calcein from double-labeled donor cells indicates activity of GJIC. Calcein: green; CM-DiI: red. Scale bar: 50 µm; n = 3. b, Quantification of gap junction assay by flow cytometry using Cell Tracker Red to mark donor cells. Double-labeled donor cell populations appear in upper-right quadrant when calcein is plotted on the x-axis and Cell Tracker Red plotted on the y-axis. Non-labeled cells appear in lower-left quadrant indicating absence of both dyes. Following 6 h co-culture of these populations at a ratio of 1:20 donor cell/acceptor cell for 6 h, calcein-only positive acceptor populations appear in the lower-right quadrant. Coupling efficiency is calculated as the number of acceptor cells divided by the number of donor cells in the experiment ± SD. (TIFF 9174 kb) [file 12079_2020_601_MOESM2_ESM.tif]

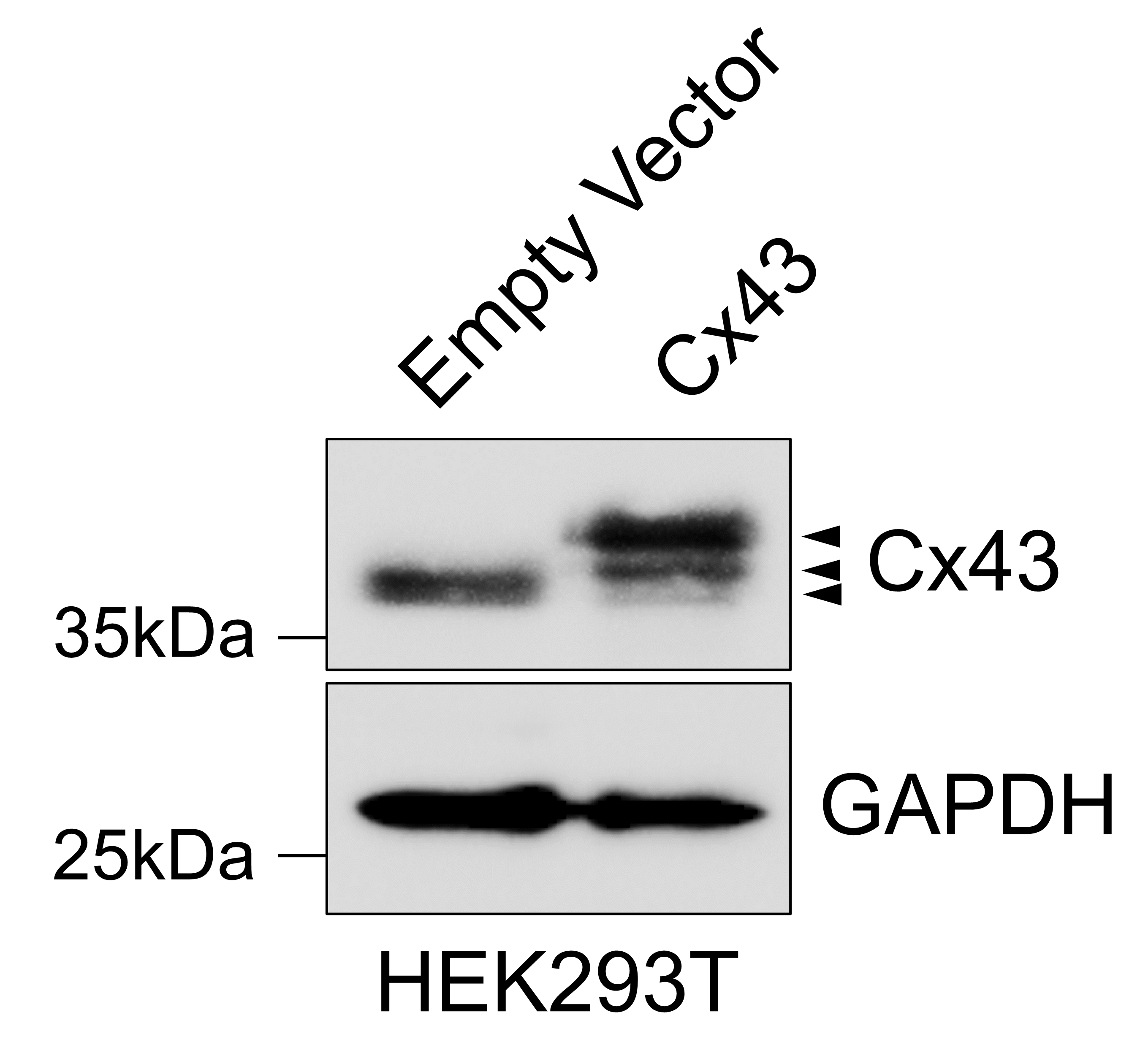

Supplement: Supplementary file 3 — Lysates of HEK293T cells with empty vector control and Cx43 overexpression were analyzed by western blot analysis. Arrows indicate multiple molecular weight bands for Cx43. GAPDH was used as a loading control. (TIFF 1486 kb) [file 12079_2020_601_MOESM3_ESM.tif]

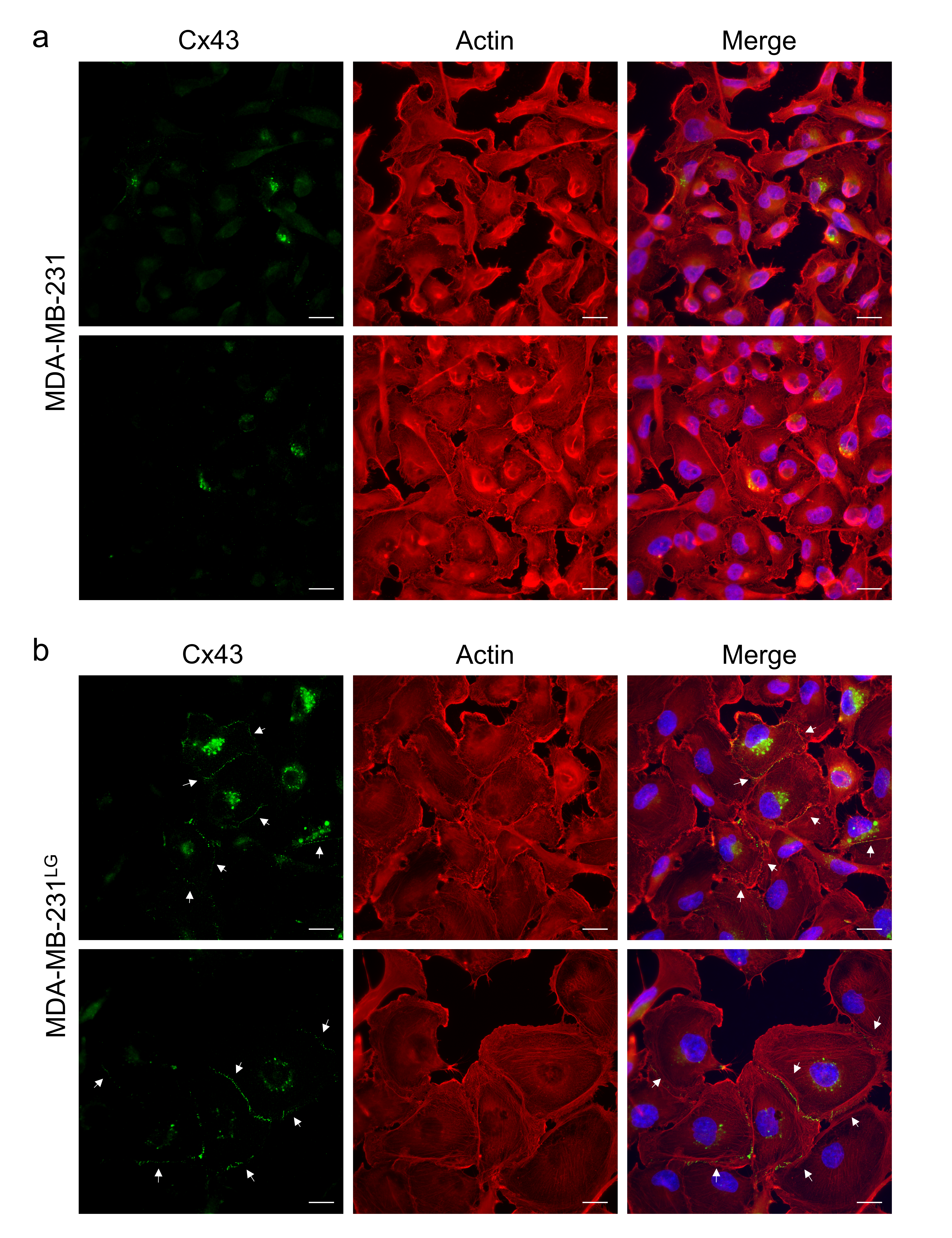

Supplement: Supplementary file 4 — Additional fields of Cx43 immunofluorescence in MDA-MB-231 (a) and MDA-MB-231LG (b) as described in Figure 4. DAPI: blue; Cx43: green; actin: red. Scale bar represents 20 µm. (TIFF 30503 kb) [file 12079_2020_601_MOESM4_ESM.tif]

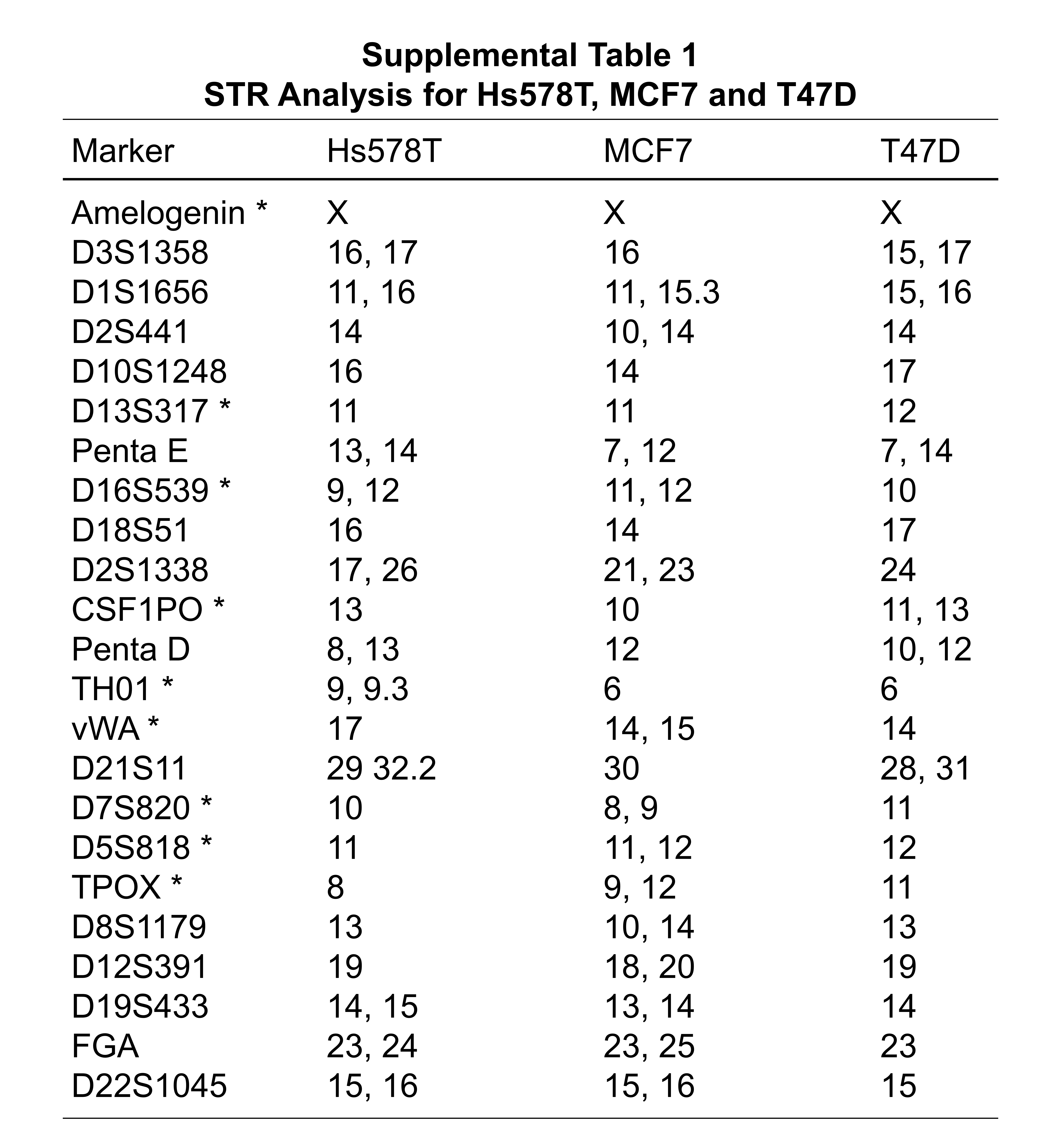

Supplement: Supplementary file 5 — STR analysis of Hs578T, MCF7 and T47D. Asterisk indicate 9 markers defined by ATCC criteria for 100% match. (TIFF 3004 kb) [file 12079_2020_601_MOESM5_ESM.tif]
